# Supplementary material for: New structural insights into the control of the retinoic acid receptors RAR/RXR by DNA, ligands, and transcriptional coregulators
Source: Nucleic Acids Res. 2025 Oct 2;53(18):gkaf967. doi: 10.1093/nar/gkaf967 (PMC12489474; doi:10.1093/nar/gkaf967)
Supplement: gkaf967_Supplemental_File [file gkaf967_supplemental_file.pdf]

SUPPLEMENTARY MATERIAL AND METHODS

RAR/RXR DBDs purification, crystallization and structure resolution of RXR/RXR DBDs in complex with IR0

The HsRXRA DBD (130-212) and HsRARA DBD (82-167) were expressed in fusion with thioredoxine and hexahistidine tags. The proteins were produced and purified as described in (1). Fusion tags were removed by thrombin proteolysis and the cleaved proteins were then purified by SEC on a Superdex 75 in 10 mM HEPES pH 8, 50 mM NaCl, 5 mM $MgCl_2$, 1 mM TCEP. The HsRXR DBD, HsRAR DBD and DNA were mixed in an equimolar ratio and concentrated to a final concentration of 7 $mg.mL^{-1}$.

To build the model of RAR/RXR bound to IR0, we attempted to crystallize the RAR/RXR DBD heterodimer assembled on IR0, but only crystals of the IR0-bound RXR DBD homodimer were obtained. The RXR/RXR IR0 crystallized in the $P4_12_12$ space group, with one dimer-DNA complex per asymmetric unit. The crystallization experiments were carried out by hanging drop vapor diffusion at 290 K by mixing equal volume (0.15 µl) of protein-DNA complex and of reservoir solution (20% PEG 1000, 0.2M MgCl2 0.1M NaCl, 0.05M Na cacodylate pH6.5). The crystals of the complex were transferred to artificial mother liquor containing 15 % Glycerol and flash cooled in liquid nitrogen. Data were collected on the Proxima 1 beamline of the synchrotron SOLEIL. The raw data were processed with XDS (2) and scaled with AIMLESS (3) programs. The structures were solved and refined using Phenix (4) and iterative model building using COOT (5). Data collection and refinement statistics are given in Table S3. The two RXR DBDs were bound to opposite sides of the DNA, and no interaction is observed between them (Figure S4-A), consistent with the binding to IR0 as two monomers at equivalent sites in a head-to-head fashion with the T-boxes of each monomer pointing towards the 3'- and 5'-flanking sequences of the IR0. Each half-site made specific contacts with 4 base pairs (Figure S4-B and C). Both RXR DBDs also formed contacts with the phosphate backbone.

Steady-State Fluorescence Spectroscopy (FRET Assay)

We performed Fluorescence resonance energy transfer (FRET) experiments to determine the spatial arrangement (polarity) of the DBDs bound to the IR0 element. The RARΔAB–RXRΔAB heterodimer was labeled via the polyHis N-terminal tag of RAR using OG488-TrisNTA (6). Free dye was removed by gel filtration. Protein concentration and labeling efficiency were estimated using the molar extinction coefficients of the protein (ε at 280 nm = 40,000 $M^{-1}·cm^{-1}$) and the dye (ε at 490 nm = 87,000 $M^{-1}·cm^{-1}$). TAMRA5/6-labeled DNA sequences were purchased from IBA GmbH (Germany). Fluorescence emission spectra were recorded using a Fluorolog or Fluoromax-3 spectrophotometer (Horiba Jobin Yvon), with excitation and emission bandwidths set to 2 nm. Measurements were acquired at 20 °C using an excitation wavelength of 470 nm (to minimize direct TAMRA excitation) and corrected for lamp fluctuations. Experiments were performed in 2 mm quartz cuvettes (Hellma GmbH). Titrations were performed by adding increasing amounts of TAMRA5/6-labeled DNA to a fixed 1 µM concentration of OG488-labeled protein (see Supplementary Figure S4 D-F). Upon complex formation, a decrease in donor fluorescence emission at 520 nm was observed, from which FRET efficiency (E) was calculated:

E = 1 – IDA / ID

where ID is the fluorescence intensity of the donor (OG488) in the absence of the acceptor, and IDA is the intensity in the presence of TAMRA5/6. Control titrations with unlabeled DNA confirmed that DNA binding did not affect the fluorescence quantum yield and lifetime of OG488 (data not shown). FRET

efficiencies measured during titration were fitted using a modified Scatchard equation, assuming a single binding site per DNA:

$$E = E_{sat} \times ((K_d + [\text{Protein}] + [\text{DNA}]) - \sqrt{(K_d + [\text{Protein}] + [\text{DNA}])^2 - 4 \times [\text{Protein}] \times [\text{DNA}]}) / (2 \times [\text{Protein}])$$

where Esat is the FRET efficiency at saturation, Kd is the dissociation constant, and [Protein] and [DNA] are the concentrations of protein and DNA, respectively. From Esat, the interchromophore distance (R) was calculated using the Förster relationship:

$$R = R_0 \times (1/E_{sat} - 1)^{(1/6)}$$

R_0 is the Förster radius, estimated using:

$$R_0 = (8.8 \times 10^{-25} \times \kappa^2 \times n^{-4} \times QD \times JAD)^{(1/6)}$$

where n is the refractive index of the medium (n = 1.33), QD is the quantum yield of the donor, and JAD is the spectral overlap integral between donor emission and acceptor absorption. Given that OG488 and TAMRA5/6 are attached via flexible linkers, an orientation factor κ^2 of 2/3 was used, assuming complete isotropic averaging.

SDS-PAGE analysis

Depending on the samples, SDS-PAGE was performed under denaturing conditions using Bis-Tris or Tris-Acetate Mini gels (Biorad, 8 cm × 8 cm, 1.0 mm thick). Gels were run in MOPS or Tris-Acetate SDS running buffers, respectively. Electrophoresis was carried out at 21.25 V/cm for 35 minutes for Bis-Tris gels or 18.75 V/cm (150 V constant) for 1 hour for Tris-Acetate gels. Gels contained 10% or 12% acrylamide as specified. Gels were stained with Instant Blue (Gentaur France) and imaged for analysis.

Size-Exclusion Chromatography Coupled to Multi-Angle Light Scattering (SEC-MALS)

SEC-MALS experiments were performed on an HPLC system (Agilent™ Infinity II) coupled to a miniDAWN TREOS multi-angle light scattering detector (Wyatt Technology™) and an Optilab T-rEX refractive index detector (Wyatt Technology™). Proteins were loaded onto a Superdex 200 Increase 10/300 GL column (GE Healthcare) pre-equilibrated in running buffer composed of 20 mM Tris-HCl (pH 7.5), 75 mM NaCl, 75 mM KCl, 2 mM CHAPS, 4 mM $MgCl_2$, and 2 mM TCEP. Analyses were performed at room temperature with a flow rate of 0.5 mL/min, and 25 µL of protein sample (typically at a concentration of 2-10 mg/mL) was injected. Light scattering and refractive index signals were recorded and used to calculate the molecular weight of the eluted species using ASTRA software (Wyatt Technology). The calculation relies on the combined use of the multi-angle light scattering signal and the concentration (from RI) to derive the molecular weight, using a known refractive index increment (dn/dc of 0.185 ml/g) for the protein sample. This approach allows molar mass determination independently of the shape or conformation of the particles. The technique's principles and its application to protein characterization can be found detailed by Some and colleagues (2019) (7).

SUPPLEMENTARY TABLES

Table S1. List of DNA sequences used in this study.

Element	Gene	Sequence	DNA length (base pair)
**DR0**	Hoxb13	5’-gaAGGTCAAGGCCAag-3’ / 3’-ctTCCAGTTCCGGTtc-5’	16
**DR1**	Idealized	5’-ctAGGTCAaAGGTCAgc-3’/ 3’-gcTGACCTtTGACCTag-5’	17
**DR5**	Rarb2	5’-agGGTTCAccgaaAGTTCAct-3’/ 3’-tcCCAAGTggcttTCAAGTga-5’	21
**IR0**	Trim16	5’- gcaGGGTCATGACCCcgc-3’/ 3’-gtCCCAGTACTGGGgcgc-5’	18
**DR0L**	Hoxb13	5’-cttccaggaAGGTCAAGGCCAagttgaaag-3’/ 3’-ctttcaactTGGCCTTGACCTtcctggaag-5	30

Table S2. Estimated parameters from SAXS intensities measured on RAR/RXR heterodimer bound to the different DNA binding sites (DR0, DR0L, DR1, DR5, IR0) in the presence and absence of $NCOR_{NID}$.

Sample	Rg (nm)		Estimated MM (kDa)		Dmax (nm)	MMseq (kDa)	Ensemble fit to data (χ2)
	Guinier	*P(r)* function	Bayesian	Credibility Interval			
RR-DR0	3.6± 0.03	3.7± 0.02	78	73-84	12.7	87	1.0
RR-DR0L	4.1 ± 0.02	4.2± 0.1	79	73-84	13.9	95	1.4
RR-DR1	3.8 ± 0.00	3.9 ± 0.06	86	84-95	12.4	88	1.4
RR-DR5	3.9 ± 0.04	4.0 ± 0.02	86	84-95	12.7	90	1.0
RR-IR0	3.9 ± 0.01	3.9 ± 0.07	83	75-86	12.6	78	1.3
NRR-DR0L	5.4 ± 0.03	5.5± 0.4	208	176-221	20.1	126	1.6
NRR-DR1	5.6 ± 0.03	6.0 ± 0.3	170	151-176	21.7	118	2.1
NRR-DR5	5.7 ± 0.06	6.0 ± 0.4	186	162-194	21.7	120	1.5
NRR-IR0	5.5 ± 0.04	6.0 ± 0.5	186	162-194	22.9	108	1.3

Table S3. Time-resolved fluorescence parameters of the RAR-RXR-OG488 in the absence and in the presence of saturating concentrations of the different DNA-TAMRA5/6. FRET efficiency at saturation (E) was calculated as described in (1).

RAR/RXR	E
RARb2 DR5*	0.46
Ramp2 DR1*	0.75
Hoxb13 DR0*	0.81
Trim16 IR0	0.69

Table S4. X-ray crystallography data collection and refinement statistics.

	**RXR DBD - IR0**
**Data collection**	
Beamline	PX1
Space group	P 4_1 2_1 2
Unit-cell parameters (Å, °)	56.019 56.019 169.871, 90.00 90.00 90.00
Resolution range (Å)	33.84-3.5
Unique reflections	3712
$CC_{1/2}$	1
Completeness	96.50
**Refinement**	
Resolution range (Å)	33.84-3.5
R_{work} / R_{free} (%)	26.53 / 33.5
Number of non-hydrogen atoms	
macromolecules	
ligands	1684
	4

SUPPLEMENTARY FIGURES

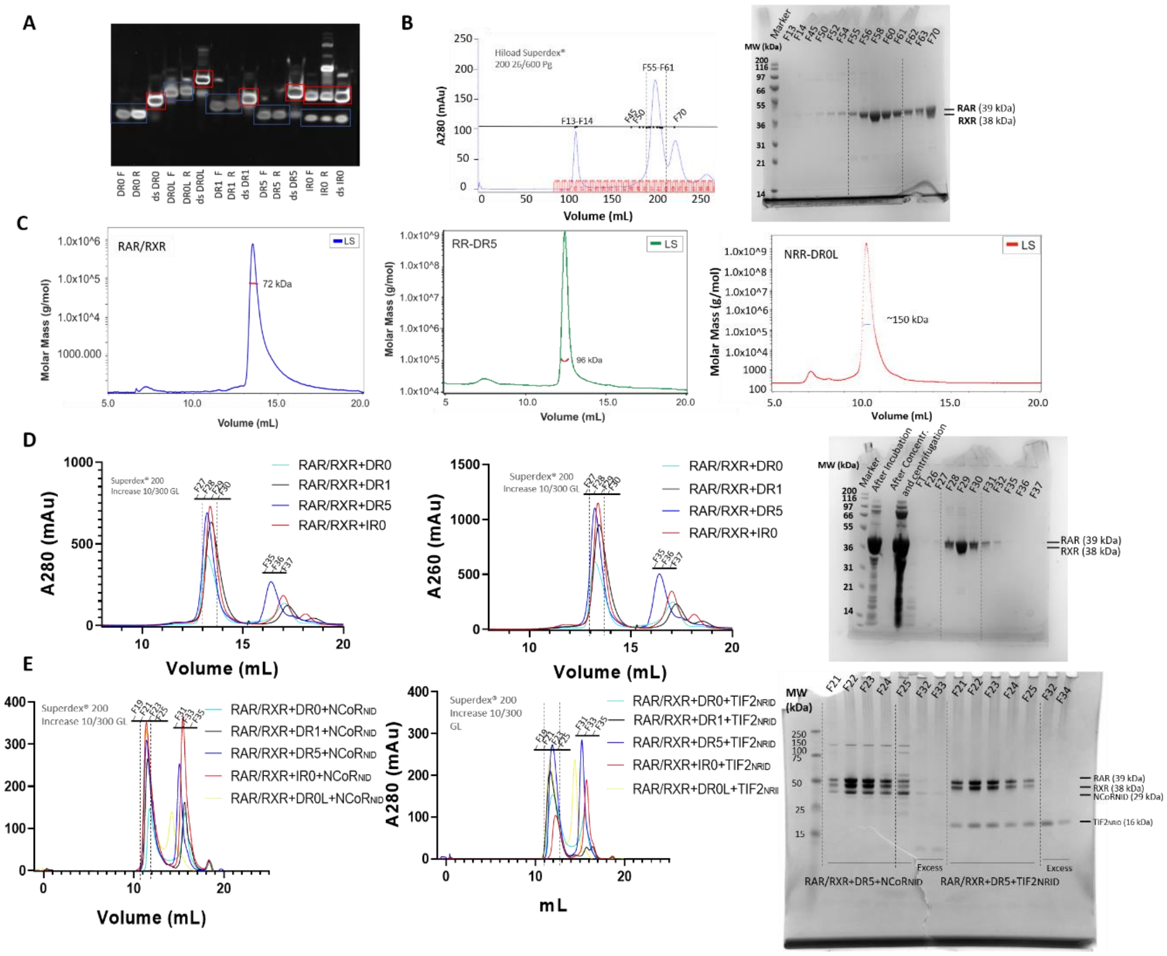

Figure S1. Sample preparation steps. A) Electrophoretic mobility shift assay (EMSA) showing the DNA single-strand migration for the forward (F) and reverse (R) sequences (in blue squares) and the band shift for the double-strand (ds) DNA after the hybridization step (in red squares). **B)** Size exclusion chromatography (SEC) chromatogram of RAR/RXR followed by the 10% SDS-page Bis-Tris gel containing the representative fraction from some aggregated sample from the first peak containing the fractions 13 and 14 (F13-F14), the heterodimer in the stoichiometry of 1:1 (second peak, F55 to 61) followed by an excess of RAR monomer (third peak, F70) due to its incubation in excess 2:1 in order to favor the heterodimer formation. The fractions (represented as "F") collected during SEC elution are identified on the SDS-PAGE gel accordingly. **C)** SEC-MALS chromatogram showing the scattered light profile for the heterodimer alone (RAR/RXR, left), the heterodimer bound to DR5 (RR-DR5, middle) and the repressive complex (NRR-DR5, right). **D)** SEC chromatogram of RAR/RXR bound to the different DNAs (following absorbance at 280 and 260 nm). The complex eluted in the first peak (F21 to F25, from the purification with RR-DR5 which is the sample loaded within the SDS-PAGE gel on the right). The dotted line represents the fractions pooled and used for further analysis.The second peak (F31 to F35) observed in the chromatogram refers to the excess of DNA added during previous incubation. The variation in the elution volume reflects the slight difference in the DNA length. The sharp peak in the expected elution volume (13 mL - by using a Superdex 200 Increase 10/300) together with the 10% SDS-page gel (fractions are identified according to their loading order in the gel during RR–DR5 purification, and their corresponding positions are indicated in parallel on the chromatogram)confirms the homogeneity and purity of the sample. **E)** SEC chromatography RAR/RXR bound to four different DNAs (DR0, DR1, DR5 and IR0) and the corepressor $NCoR_{NID}$ (left). The elution volume (~11 mL, F21 to F24 as using the NRR-DR5 purification as reference) reveals the shift provoked by corepressor binding. SEC chromatography RAR/RXR bound to the different DNA elements and the coactivator TIF-2_{NRID} (center). The elution volume (~11.5 mL, F21 to F25 as using the TRR-DR5 purification as reference) reveals the shift caused by the complex formation with the coactivator. SDS-PAGE Tris-Acetate gel (right), providing decent resolution for 39, 38, 29 and 16 kDa proteins, confirming the complexes formation. The fractions are labeled according to their loading sequence on the SDS-PAGE gel from the NRR-DR5 and TRR–DR5 purification, with their corresponding elution positions simultaneously indicated on the chromatograms.The similar intensity of bands confirms the stoichiometry of the complexes (in agreement with SEC elution volume and SEC-MALS molecular weight estimation) and its purity.

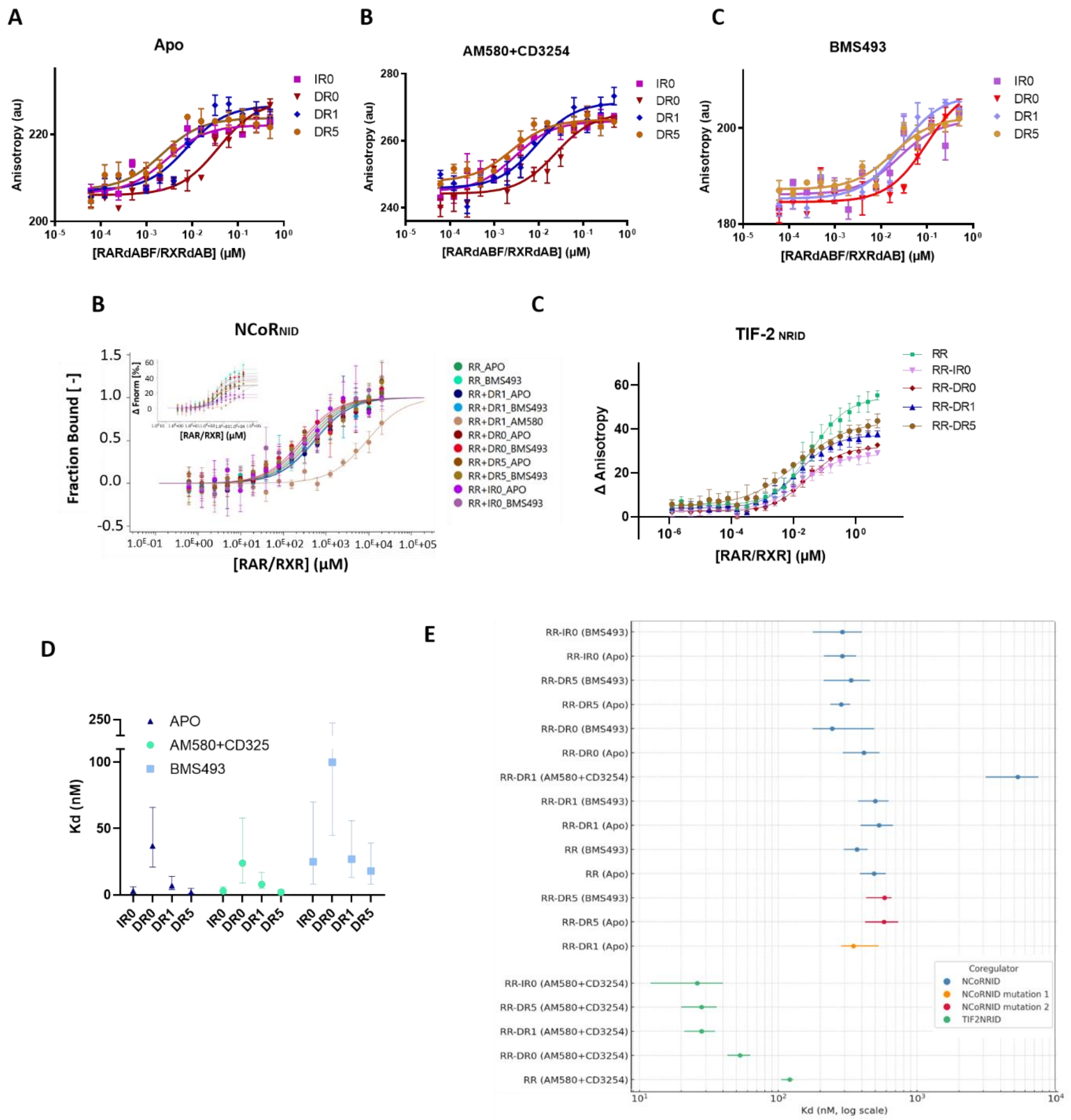

Figure S2. Titration curves of the affinity measurements on RAR/RXR heterodimer. A) Affinity curves obtained by fluorescence anisotropy for heterodimer RAR/RXR and the different DNAs (IR0, DR0, DR1 and DR5) in the absence of the ligand (left), or in the presence of the agonists AM580 and CD3254 (middle), or the presence of the inverse agonist BMS493 (right). Fluorescence anisotropy binding curves were fitted using a sigmoidal dose–response model with variable slope using Prism Graphpad. **B)** Affinity curves measured by microscale thermophoresis of the RAR/RXR unbound (RR) and bound to the different DNAs (RR-IR0, RR-DR0, RR-DR1, RR-DR5) with the corepressor $\mathrm{NCoR_{NID}}$, in the presence and absence (apo) of the inverse agonist BMS493. **C)** Affinity curves by fluorescence anisotropy for the RAR/RXR unbound (RR) and bound to the different DNAs (RR-IR0, RR-DR0, RR-DR1, RR-DR5) with the coactivator $\mathrm{TIF\text{-}2_{NRID}}$. All the measurements were performed in the presence of the RARα and RXR respective agonists, AM580 and CD3254. **D)** Kd values measured between the RAR/RXR heterodimer and the different DNA elements (DR0, DR1, DR5 and IR0) in the absence and the presence of the RAR inverse agonist and/or the RAR (AM580) and RXR agonist (CD3254), as given in Table 1. **E)** Kd values measured for the coregulators with the RR-DNA complexes. Error bars represent confidence ranges calculated according to the affinity measurement technique. Color coding indicates the coregulator involved ($\mathrm{NCoR_{NID}}$ in blue, $\mathrm{NCoR_{NID}}$ mutant 1 in orange, $\mathrm{NCoR_{NID}}$ mutant 2 in red, $\mathrm{TIF\text{-}2_{NRID}}$ in green). All the reported data are the average of at least three independent experiments.

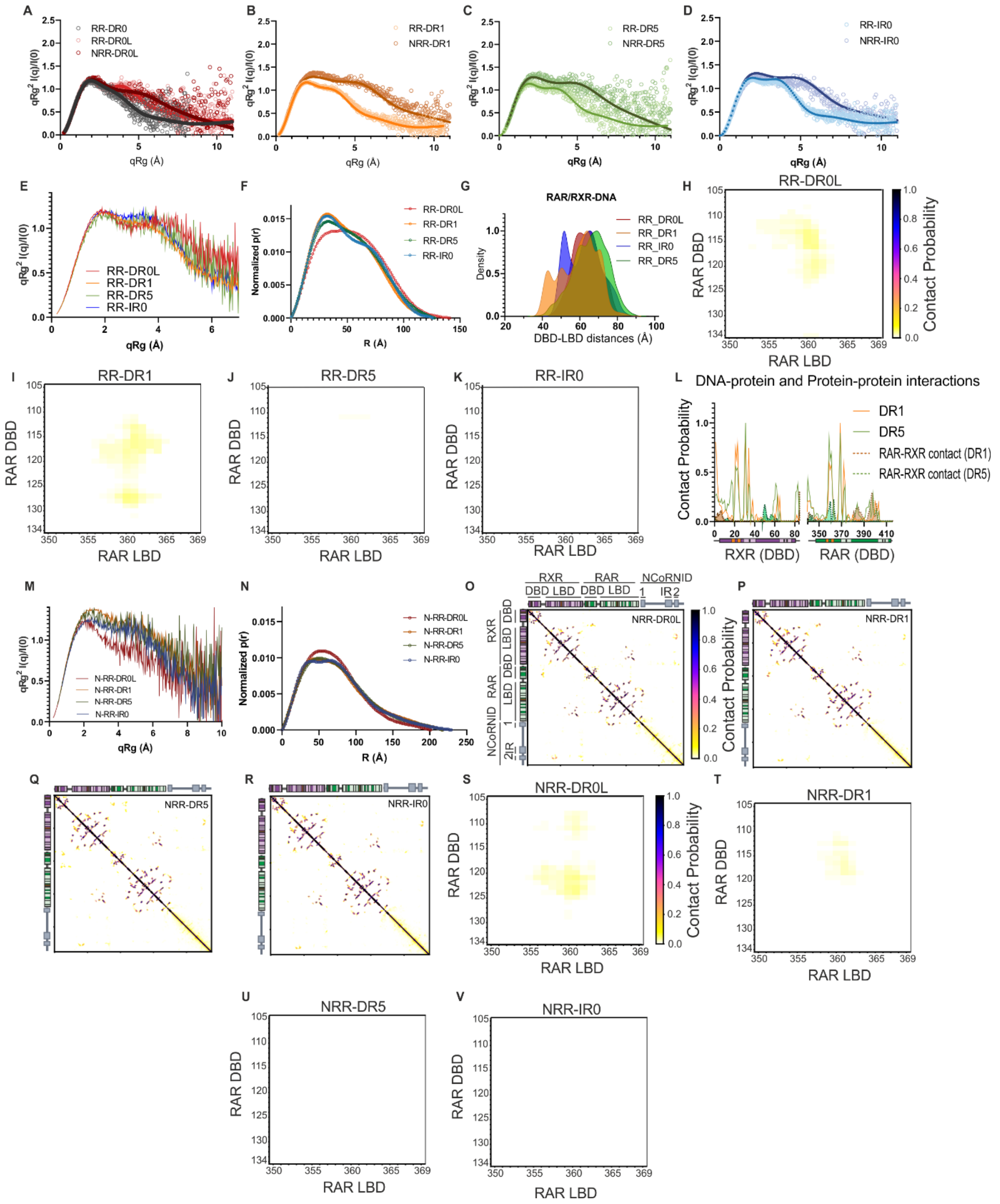

Figure S3. SAXS and molecular dynamics analysis of RAR/RXR complexes bound to different DNA binding sites in the presence and absence of NCoR$_{NID}$. (A-D) Kratky plots derived from SAXS for RAR/RXR complexes bound to the different DNA binding sites in the absence (RR-DNAs) and presence of NCoR$_{NID}$ (NRR-DNAs). Panel (A) shows RR-DR0 (grey), RR-DR0L (red), and N-RR-DR0L (dark red). Panel (B) depicts RR-DR1 (orange) and N-RR-DR1 (dark orange); panel (C) displays RR-DR5 (green) and N-RR-DR5 (dark green); and panel (D) presents RR-IR0 (blue) and NRR-DR0 (dark blue). **(E)** Superimposed Kratky plots for RR complexes bound to the distinct DNA binding sites (DR0L, DR1, DR5, IR0). **(F)** Superposition of pairwise distance distribution functions P(r) for RR-DNA complexes. **(G)** Superposition of distances calculated between the centers of mass of the DBD and LBD for RR-DNA complexes. **(H-K)** Contact probability submatrix for the RAR DBD and LBD regions (ranging from 105-134 and 350-369 residues, respectively) showing the transient interdomain contacts on RAR bound to DR0L and DR1 predominantly. Panels correspond to the respective DNA binding sites: (H) DR0L; (I) DR1; (J) DR5; (K) IR0. **(L)** Contacts between the DBDs of RAR and RXR with DNA in RR complexes bound to DR1 (yellow) and DR5 (green). The dotted lines indicate inter-subunit DBD interactions: dark yellow for DR1 and green for DR5. **(M)** Superimposed Kratky plots for NRR-DNA

complexes. **(N)** Superposition of P(r) plots obtained for NRR-DNA complexes. **(O-R)** Contact probability maps for NRR-DNA complexes from the molecular dynamics simulations after bayesian re-weighting. Proteins' secondary structures are indicated by colored bars: RXR (purple) and RAR (green), and NCoRNID (lilac), with alpha-helices (light shades) and beta-sheets (orange). Panels correspond to the DNA binding sites: DR0L (O) DR1 (P), DR5 (Q), and R0 (R). **(S-V)** Contact probability submatrix for the RAR DBD and LBD regions (ranging from 105-134 and 350-369 residues, respectively) within the NRR-DNA complexes showing the transient interdomain contacts on RAR bound to DR0L and DR1 predominantly. Panels correspond to the respective DNA binding sites: (S) DR0L; (T) DR1; (U) DR5; (V) IR0.

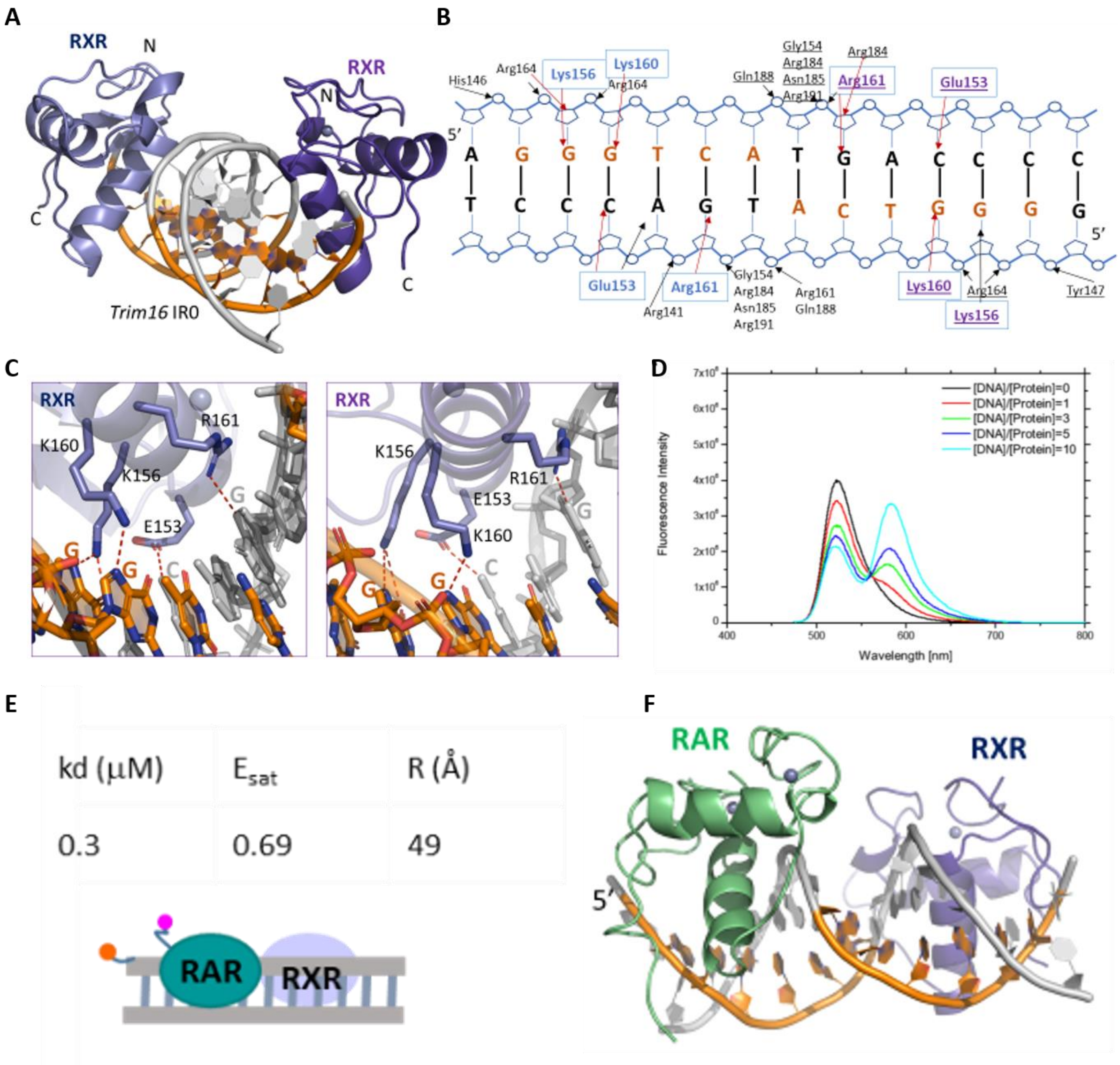

Figure S4. Crystal structure of RXR–RXR DBDs-Trim16 IR0 and polarity of the RAR/RXR DBDs bound to Trim16. (A) Overall structure of RXR–RXR–DNA complex. The spheres indicate the Zn atoms. **(B)** Schematic view of the RXR–RXR DBDs-Trim16 IR0 contacts calculated with NUCPLOT with a 3.9 Å distance cutoff. **(C)** Specific interactions of RXR homodimer DBDs to Trim16 IR0. Left: View along the DNA-recognition helix of 5' RXR. Right: The corresponding view of 3' RXR. Hydrogen-bonds are shown as red dotted lines. **(D)** Fluorescence spectra of OG488-RARADAB-RXRADAB in the absence (black curve) and in the presence of increasing concentrations of TAMRA5/6-Trim16 IR0 (red, green, blue and cyan curves correspond respectively to DNA/protein ratios of 1, 3, 5 and 10). The FRET efficiency was calculated from the decrease of the donor emission. **(E)** Calculated parameters for the fitting of FRET efficiency on DNA concentration. The affinity (kd) is similar to previously measured for Ramp2 DR1 (kd = 0.5 M) and Rarb2 DR5 (kd = 0.8 M) (1). FRET efficiency at saturation (Esat ~0.7) suggest a close averaged distance between the two dyes, similar Ramp2 DR1 complex (Esat=0.75) and larger than on Rarb2 DR5 (Esat =0.46) (1), in agreement with a binding mode on Trim16 IRO similar than on DR1 with RAR positioned on the 5' half-site. Red and orange spheres correspond to the location of the dyes. **(F)** 3D model of RAR-RXR DBDs bound to Trim16 IR0 with RAR bound to the 5' half site based on time-resolved fluorescence parameters.

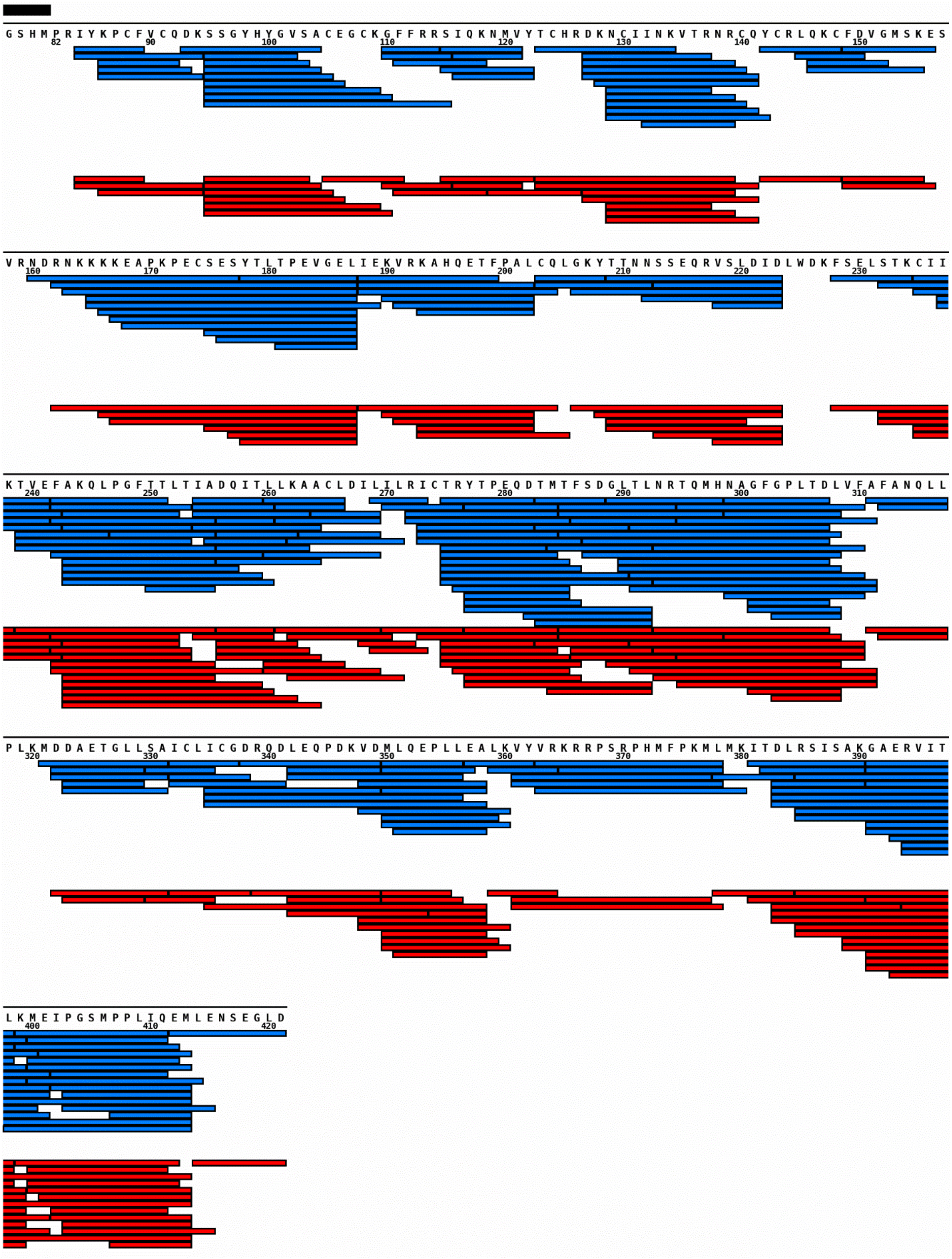

Figure S5. Sequence coverage of RAR. Coverage map of RAR in the experiment with TIF-2NRID (blue bars) and NCoRNID (red bars). Digestion parameters for TIF-2/NCoR data were - sequence coverage: 93.4%/92.6%. Number of peptides: 210/147. Average peptide length: 11.7/11.7. Average redundancy: 7.5/5.3. Only peptides providing HDX data are shown. The black box highlights the sequence from the tag. The map was plotted using MSTools (https://peterslab.org/MSTools/DrawMap/DrawMap.php) (8).

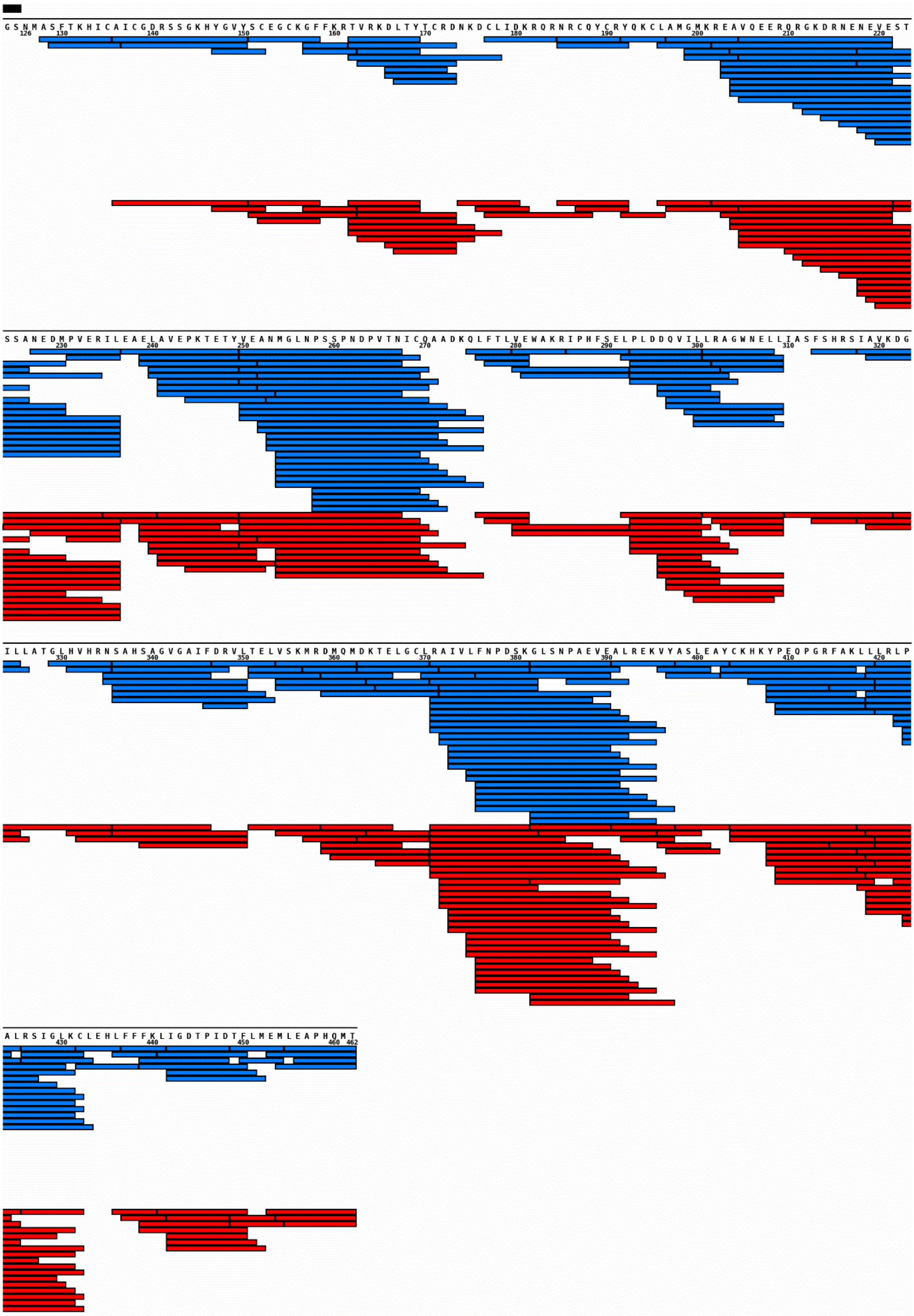

Figure S6. – Sequence coverage of RXR. Coverage map of RXR in the experiment with TIF-2NRID (blue bars) and NCoRNID (red bars). Digestion parameters for TIF-2/NCoR data were - sequence coverage: 95.4%/93.6%. Number of peptides: 210/192. Average peptide length: 12.6/12.5. Average redundancy: 8.0/7.4. Only peptides providing HDX data are shown. The black box highlights the sequence from the tag. The map was plotted using MSTools (https://peterslab.org/MSTools/DrawMap/DrawMap.php) (8).

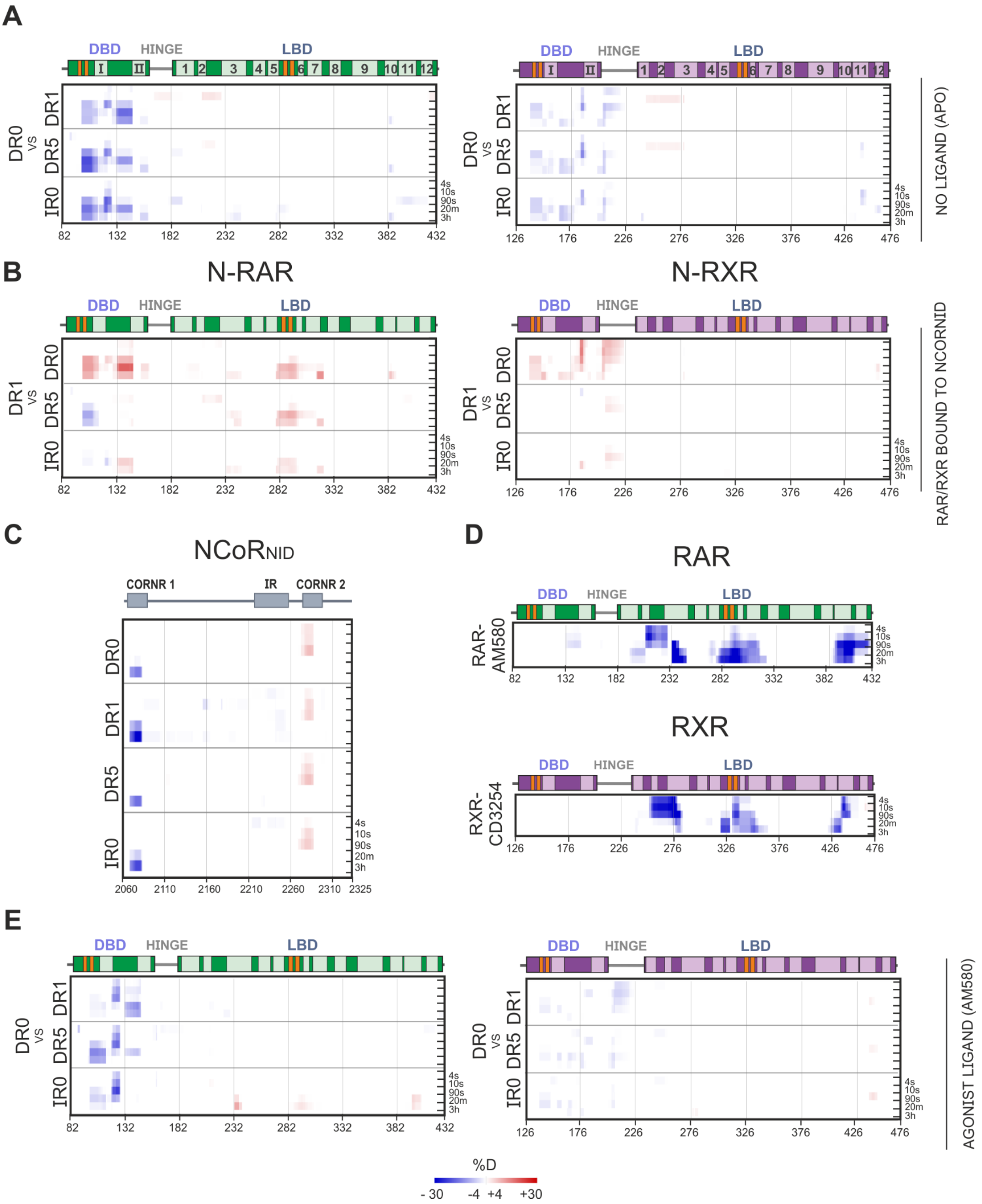

Figure S7. Further details on the differential protection on RAR/RXR heterodimer and NCoRNID caused by the DNA and ligands. (A) Heatmap of the apo RAR (left) and RXR (right) showing the differential in protection between DR0 and the other DNA conditions, such as bound to DR1 (DR1 - DR0), DR5 (DR5 - DR0) and IR0 (IR0 - DR0). Red and blue shades represent increased and decreased deuterium exchange, respectively. The α-helices are represented in lighter-colored rectangles followed by their respective numbering. β-strands are represented in orange. **(B)** Heatmap of the RAR (left) bound to the inverse agonist and corepressor (left) and apo RXR in the presence of the corepressor (right) showing the differential in deuterium incorporation caused by the DR1 binding compared among the other DNA binding sites. **(C)** Differential HDX-MS heatmap analysis on NCoR$_{NID}$ showing the DNA-ligand effect on increasing the protection on its CoRNR1 and decreased on the CoRNR2. **(D)** Heatmaps showing the protection caused by the agonist ligand AM580 interaction with the RAR (upper) and RXR (lower). **(E)** Differential heatmap of the agonist bound RAR (AM580, left) and RXR (CD3254, right) showing the variation in protection between DR0 and the other DNA conditions, such as bound to DR1 (DR1 - DR0), DR5 (DR5 - DR0) and IR0 (IR0 - DR0).

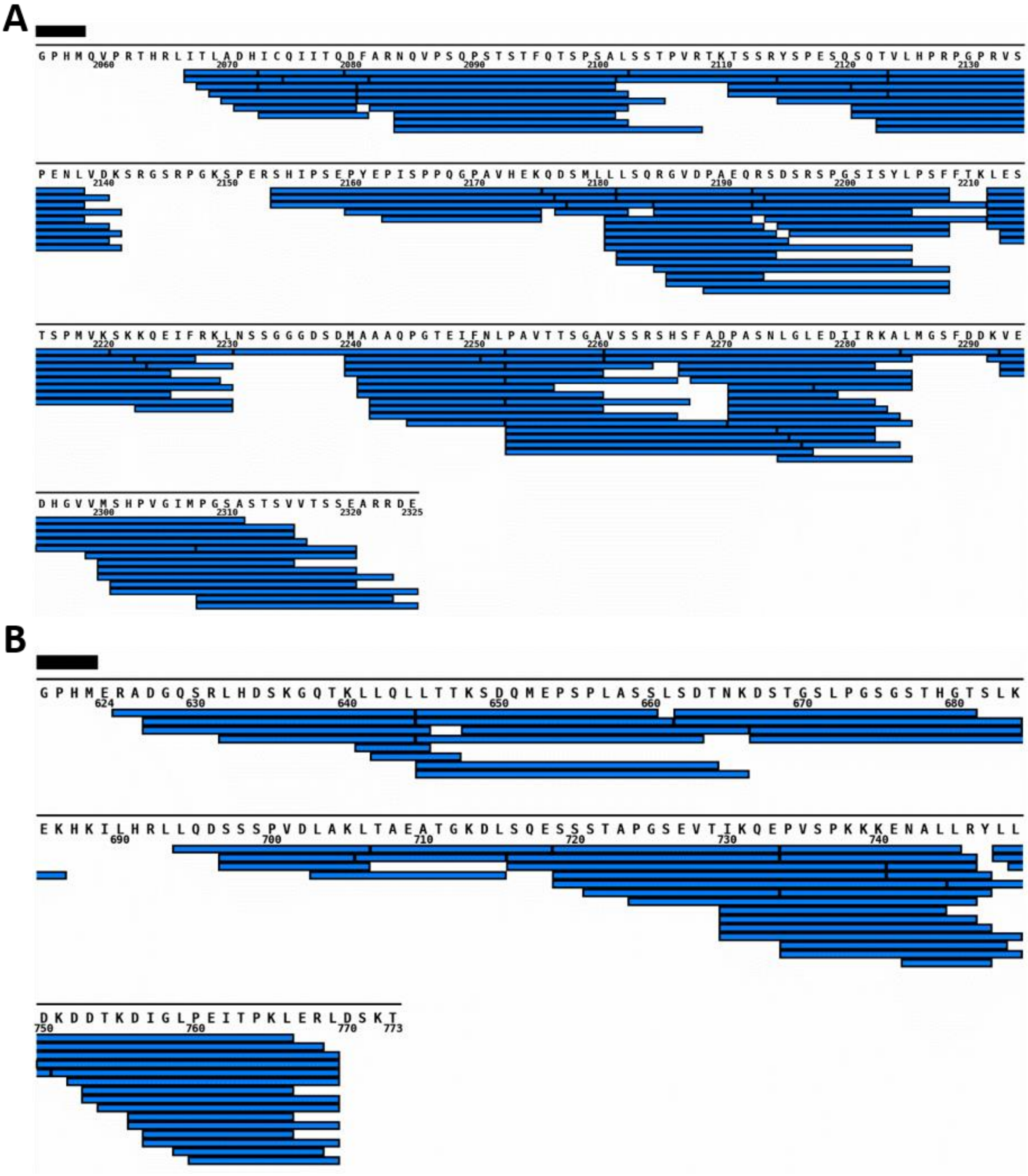

Figure S8. Sequence coverage of NCoRNID and TIF2 NRID. Coverage map of NCoRNID **(A)** and TIF-2 NRID **(B).** Digestion parameters for NCoR were - sequence coverage: 89.54%, number of peptides: 127, average peptide length: 15.5, average redundancy: 8.0. Digestion parameters for TIF-2 were - sequence coverage: 87.8%, number of peptides: 57, average peptide length: 15.5, average redundancy: 6.4. Only peptides providing HDX data are shown. The black box highlights the sequence from the tag. The map was plotted using MSTools (https://peterslab.org/MSTools/DrawMap/DrawMap.php) (8).

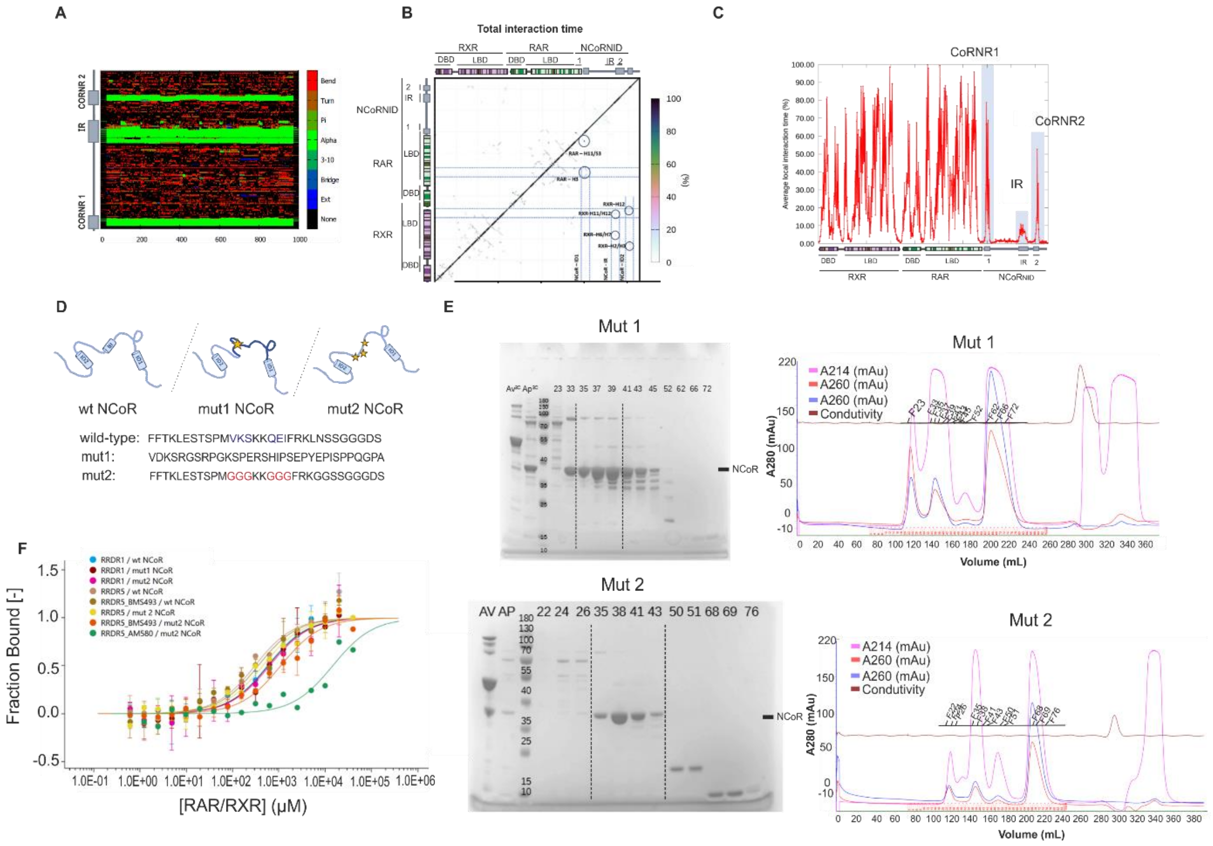

Figure S9. NCoR$_{NID}$ mutants sample preparation and affinities with the RR-DNA complexes. A) Secondary structure proportion of the NRR-DR1 complex through molecular simulation. **B)** Contact map among the proteins in the NRR-DR1 complex. **C)** Average local interaction time (%) within the NRR-DR1. **D)** Illustration of the two mutations designed for NCoR$_{NID}$ on its intermediate region (IR). NCoR$_{NID}$mut1 is composed of a complete replacement of the IR on the NCoR$_{NID}$. NCoR$_{NID}$mut2 is composed of punctual mutations to glycines on crucial points for IR helicity, potentially causing the disruption of its secondary structure as well as its motif of interaction with the heterodimer. **E)** SDS-PAGE analysis following the final SEC purification step for NCoRNIDmut1 (top) and NCoRNIDmut2 (bottom), illustrating the purity of both samples. The chromatograms are displayed on the right, with annotated fractions directly corresponding to the lanes loaded on the gels (left). Sample labels follow the convention: 'AV' indicates fractions collected before 3C protease cleavage, and 'AP' denotes those after cleavage. The numbers indicate each fraction loaded into its respective well, matching its position on the chromatogram. Dotted lines indicated the fractions that were pooled and next concentrated for further analysis.**F)** Affinity curves measured by microscale thermophoresis of the RR-DNA complexes with the corepressor NCoR$_{NID}$mut1 and 2, in the presence and absence (apo) of the inverse agonist BMS493 or the RARα agonist (AM580).

SUPPLEMENTARY BIBLIOGRAPHY

1. Osz,J., McEwen,A.G., Bourguet,M., Przybilla,F., Peluso-Iltis,C., Poussin-Courmontagne,P., Mély,Y., Cianférani,S., Jeffries,C.M., Svergun,D.I., *et al.* (2020) Structural basis for DNA recognition and allosteric control of the retinoic acid receptors RAR–RXR. *Nucleic Acids Res*, **48**, 9969–9985.
2. Kabsch,W. (2010) XDS. *Acta Crystallogr D Biol Crystallogr*, **66**, 125.
3. Evans,P. (2005) Scaling and assessment of data quality. *urn:issn:0907-4449*, **62**, 72–82.
4. Adams,P.D., Afonine,P. V., Bunkóczi,G., Chen,V.B., Davis,I.W., Echols,N., Headd,J.J., Hung,L.W., Kapral,G.J., Grosse-Kunstleve,R.W., *et al.* (2010) PHENIX: a comprehensive Python-based system for macromolecular structure solution. *Acta Crystallogr D Biol Crystallogr*, **66**, 213–221.
5. Emsley,P., Lohkamp,B., Scott,W.G. and Cowtan,K. (2010) Features and development of Coot. *Acta Crystallogr D Biol Crystallogr*, **66**, 486–501.
6. Lata,S., Gavutis,M., Tampé,R. and Piehler,J. (2006) Specific and stable fluorescence labeling of histidine-tagged proteins for dissecting multi-protein complex formation. *J Am Chem Soc*, **128**, 2365–2372.
7. Some,D., Amartely,H., Tsadok,A. and Lebendiker,M. (2019) Characterization of proteins by size-exclusion chromatography coupled to multi-angle light scattering (Sec-mals). *Journal of Visualized Experiments*, **2019**.
8. Kavan,D. and Man,P. (2011) MSTools—Web based application for visualization and presentation of HXMS data. *Int J Mass Spectrom*, **302**, 53–58.
